# Sustainability of Interprofessional Education: Protocol for a Scoping Review

**DOI:** 10.2196/60763

**Published:** 2024-12-09

**Authors:** Nathalia Hanany Silva de Oliveira, Renata Fonsêca Sousa de Oliveira, Juliana Pontes Soares, Janete Lima de Castro

**Affiliations:** 1 Department of Collective Health Federal University of Rio Grande do Norte Natal Brazil; 2 Health Human Resources Observatory Federal University of Rio Grande do Norte Natal Brazil; 3 Insikiran Institute Federal University of Roraima Boa Vista Brazil

**Keywords:** college education, health training, interprofessional education, IPE, sustainability, collaborative practice, training of human resources in health, undergraduate medical education, students, student learners, professional development, health care professional training

## Abstract

**Background:**

Interprofessional education (IPE) is an approach that can improve health care quality, contribute to the qualification of health care professionals, and train undergraduate students. Although this strategy has made significant progress in the last decade, integration, sustainability, and institutional growth are still priorities worldwide. Thus, maintaining strategies is essential for their full development and evolution.

**Objective:**

This study aimed to identify discussions about the sustainability of IPE and map its actions or strategies (or both).

**Methods:**

The scoping review will follow the Joanna Briggs Institute methodology. This scoping review protocol follows the *JBI Reviewers’ Manual*, with 6 stages: identifying the research question; identifying relevant studies; study selection; data extraction and coding; analysis and interpretation of results; and consultation with stakeholders. Two independent and blind reviewers will evaluate and select studies available in English, Portuguese, and Spanish based on the eligibility criteria. Searches will be conducted on LILACS, Embase, Scopus, PubMed/MEDLINE, ERIC, Web of Science, CINAHL, Google Scholar databases; ProQuest Dissertations & Theses Global, and Brazilian Digital Library of Theses and Dissertations. The main research question is as follows: What have been the sustainability strategies for IPE actions? This scoping review will incorporate studies (empirical or theoretical-reflective) that address strategies or actions (or both) for IPE sustainability. They must present a quantitative, qualitative, or mixed methods approach and be available in full text. Data on strategies or actions for IPE sustainability will be extracted and inserted into a spreadsheet for analysis. Quantitative data will be analyzed using descriptive statistics, while qualitative analysis will identify meanings and patterns through thematic analysis. Thus, the aim is to present the compiled findings in tables and charts.

**Results:**

The database search was conducted on March 22, 2024. In April and May 2024, duplicate studies were excluded. From July to November 2024, study selection will be carried out. In December 2024, data extraction and tabulation will take place, as well as consultation with stakeholders. The aim is to publish the results in scientific journals in January 2025.

**Conclusions:**

This protocol will guide this scoping review to identify discussions on the sustainability of IPE and map its actions or strategies (or both); summarize the definitions and institutions that develop or promote IPE; and present the main recommendations for the area under study. Additionally, possible research gaps can be identified to guide future studies. This review will shed light on existing knowledge gaps and the current state of research, which could provide support for future research, programs, and policy responses to foster collaboration and interprofessional practice and, consequently, improve the quality of user care. This information will be useful in supporting decision-making by government officials, managers, teachers, facilitators, and students in the implementation, maintenance, and development of IPE.

**Trial Registration:**

Open Science Framework 5VNJS; https://osf.io/5vnjs/

**International Registered Report Identifier (IRRID):**

DERR1-10.2196/60763

## Introduction

The current social and economic situation is changing the epidemiological and demographic profile worldwide, increasing social inequalities and health care problems. Therefore, changes are needed in health systems and the training of health care professionals [1]. In this context, interprofessional education (IPE) qualifies health care professionals and trains students from several undergraduate programs, evidencing its ability to improve health care [2]. This strategy allows members of more than 1 health profession to interactively learn how to improve interprofessional collaboration or the health and well-being of patients [3]; the latter is one of the most important benefits of IPE [4].

The literature also highlights the benefits of IPE to students, facilitators, and patients of health services. Students benefit from an improved understanding of other health professions, as well as practical learning on how to work in interprofessional teams and maximize patient care and outcomes. For facilitators, IPE provides reflections and learning about their professional practice and highlights the importance of the entire team focusing on the patient care. For patients, IPE improves the quality of care [5]. IPE starts from recognizing that the health-disease process is the expression of life and work (ie, how individuals, families, and social groups are inserted in society) and meeting the needs of the complex health care network, based on collaboration between health care professionals [6].

Global changes, especially after the COVID-19 pandemic, have presented an opportunity to increase innovation in education, health systems, and their interaction. In this sense, education facilitated by information technologies began to be used, offering subsidies for competency-based education and IPE [7]. Although this strategy has made significant progress over the last decade, the Interprofessional Research Global stated that IPE integration, sustainability, and growth at the institutional level are still priorities worldwide [8]. Therefore, maintaining strategies is essential for its full development and evolution.

Thus, this study understands sustainability as strategies, elements, initiatives, or investments (or both) that contribute to and ensure the continuity of IPE at the macro, meso, and micro levels [9,10].

Several barriers affect the performance of IPE, such as the hegemonic trend of uniprofessional training; the management of health services and its influence on the organization of the work process in health in a productive sense (quantity of procedures); the significant turnover of team members; and, primarily, the COVID-19 pandemic, identified as the greatest obstacle to the successful implementation of this interprofessional initiative [9]. Therefore, sustainability for IPE remains a persistent challenge due to the adversities that hinder its progress.

The synthesis of sustainability strategies or actions (or both) for IPE may help managers, teachers, and facilitators in their decision-making at their institutions. The compilation may assist them in maintaining IPE in their practice settings (educational institutions or health services) and developing new sustainability strategies. Furthermore, this scoping review may assist in the development of new research.

Considering the need to synthesize and consolidate sustainability strategies for maintaining IPE and global changes after the pandemic, this scoping review will seek to identify discussions about the sustainability of IPE and map its actions or strategies (or both).

## Methods

### Overview

The proposed scoping review will be conducted in accordance with the JBI methodology [11] for scoping reviews and the PRISMA-ScR (Preferred Reporting Items for Systematic Reviews and Meta-Analyses Extension for Scoping Reviews) guidelines [12] for the presentation of the research reports. Also, the review will consider the recommendations by Peters et al [13] to ensure rigor, transparency, and reliability.

A preliminary search was conducted in January 2024, as Joanna Briggs Institute (JBI) recommended [11]. The International Prospective Register of Systematic Reviews, the Cochrane Library, JBI Evidence Synthesis, and the MEDLINE (by PubMed) databases were searched. The search terms used were (“Interprofessional education”) AND (“Sustainability” OR “Sustain” OR “Continuity” OR “Durability” OR “Sustainabilities”); and in the Virtual Health Library (“Interprofessional Education”) AND (“Sustainability” OR “Maintenance” OR “Continuity” OR “Durability”).

A literature review study [14] was identified; the authors evidenced the barriers and facilitators for IPE until December 2012. They highlighted the importance of facilitators for IPE sustainability. However, this review neither focused on sustainability nor followed the JBI recommendations, which are reliable since they ensure transparency and rigor. Thus, this topic requires an update.

### Review Question

The research question was formulated using the Population, Concept, and Context mnemonic [15].

#### Population

This review will consider studies that explore any IPE initiatives in courses, programs, electives, mandatory subjects, extensive activities, projects developed in communities, hospital training wards, care teams, common activities, online modules in the distance education modality, case discussions, and simulated activities; these interventions must have involved at least 2 health professions [16]. Thus, the population is broad [17].

#### Concept

This review will consider studies that explore IPE sustainability. Sustainability is defined as the condition or quality of something that can sustain, defend, maintain, or conserve itself [18]. In 2010, the World Health Organization launched the “Framework for Action on Interprofessional Education and Collaborative Practice” [19], which had an influence worldwide. This framework followed the changes in the education of health care professionals. The document provided strategies and ideas that helped policy makers implement IPE and collaborative practice in public policy.

In America, the Pan American Health Organization released a technical cooperation that launched a Regional Network on IPE and strengthened its implementation in national human resources policies for health. Thus, initiatives were needed to ensure IPE sustainability in the academic area, continuing health education practices, and daily teamwork [16]. For instance, Brazil launched an action plan to implement IPE, a promising initiative to ensure IPE sustainability in the country [16]. The plan includes initiatives such as curricular changes and the educational practices of health care professionals using IPE.

#### Context

This review will consider studies developed in the IPE context, whether an academic, educational, or professional practice, that included curriculum-based or continuing IPE education. No restrictions will be made regarding geographic location, sex, ethnicity, or other demographic components. IPE is a method that emerged to train health care professionals to promote collaboration within and between teams, improving health care quality [20].

In this context, the questions of this scoping review protocol were defined as follows: How is the literature presenting the discussion of IPE sustainability? Has IPE sustainability entered the agendas of teaching-service institutions? What have been the sustainability strategies for IPE actions?

### Identification of Relevant Studies

The search strategy will be formulated based on a combination of controlled descriptors (health science descriptors, Medical Subject Headings [MeSH], CINAHL subject headings, and Emtree) and keywords (or both) related to the topic, associated with the Booleans operators “AND” and “OR.”

Searches will be conducted in the LILACS, Embase, Scopus, MEDLINE, ERIC, Web of Science, and CINAHL databases. Sources of unpublished studies and gray literature will also be searched in Google Scholar, ProQuest Dissertations & Theses Global, and Brazilian Digital Library of Theses and Dissertations. A manual search will also be conducted in the reference lists of the initially selected studies to find other eligible studies. Furthermore, authors will be contacted by email to request the full text when needed.

An initial limited search was performed on the MEDLINE database to identify studies on the topic. The terms contained in the titles and abstracts of the relevant studies and their index terms were used to develop a comprehensive search strategy for MEDLINE (refer to Multimedia Appendix 1). The search strategy will be adapted for each information source, including all identified keywords and index terms. The reference lists of studies selected for full-text review will be analyzed to identify other eligible studies.

The search strategy will locate primary studies, reviews, and opinion articles. Duplicates, studies, documents whose acquisition is not allowed through university institutional log-in, and those that are out of scope will be excluded, that is, articles that do not respond to the objective of the study.

This scoping review will consider studies available in full text (empirical or theoretical-reflective) that present a quantitative, qualitative, or mixed methods approach. In addition, systematic reviews and opinion articles will be considered for inclusion. Studies published in English, Spanish, and Portuguese will be included. Those in other languages will not be included due to financial and time constraints for translations. The date of publication will not be restricted.

### Study Selection

After searching the databases, all identified records will be grouped and imported into Rayyan; duplicates will be removed. Following pilot-testing, 2 independent reviewers will assess titles and abstracts based on the inclusion criteria. Potentially relevant studies will be retrieved in full text, and their citation will be imported from Rayyan. Two independent reviewers will also assess the full text of the citations. Reasons for excluding full-text studies will be reported in the scoping review. A third reviewer will be consulted in case of disagreements between the reviewers at each stage of the study selection. The research results will be fully reported in the final scoping review and presented in a PRISMA flow diagram [21].

### Data Extraction and Coding

In a pilot test, 2 independent reviewers will extract data from the studies included using a data extraction tool (Microsoft Excel) developed by the reviewers. The pilot test will be conducted with a random sample of the first 25 studies (title and abstract), which will be organized in alphabetical order using the Rayyan software, to assess the eligibility criteria and agreement among researchers; the agreement must reach at least 75% before proceeding with independent evaluation. The reviewers will later check the extracted data to discuss discrepancies and ensure accuracy. Details about study selection (identification, screening, eligibility, and inclusion) will be presented in a flowchart.

The extracted data will include specific details about the initiatives, courses, programs, or disciplines (population); strategy or action for IPE sustainability (concept); definition that the study uses for IPE (context); characteristics of discussions on IPE sustainability; and evidence source details and results. A draft extraction tool will be used (Table 1), which was modified and revised as needed during the data extraction for each included study. Modifications will be detailed in the full scoping review. Any disagreements between reviewers will be resolved by discussion with a third reviewer. Authors will be contacted to request missing or additional data when needed. As scoping reviews do not seek to assess the quality of the selected studies, the risks of bias in the studies will not be assessed.

**Table 1:** Instrument for data extraction.

Data^a^	Description
Type of production	Study, book chapter, event summary, dissertation, thesis, and others
Research approach	Quantitative, qualitative, or mixed
Publication date	Year of publication
Country where the study was conducted	Where the study was conducted or published
Scientific journal	Journal that published the study
Author qualification	Highest title of the first author
Title	Study title
Objective/purpose	Objective or purpose of the study
Type of study	Type of research described by the authors
Data collection	Methods for data collection
Study population	Name of initiatives, courses, programs, and disciplines
Concept: sustainability	Description of the strategy or action for IPE^b^ sustainability
Context: IPE	Describe the concept the article uses for IPE
Development institution	Institution that promoted or developed strategies or actions for the IPE sustainability
Results	Main results of the study
Recommendations	Recommendations that the study can offer for IPE

^a^Adapted from the Joanna Briggs Institute model [11].

^b^IPE: interprofessional education.

### Analysis and Interpretation of Results

Quantitative data will be analyzed using descriptive statistics and presented in absolute or relative frequency, such as type of production, research approach, publication date, country where the study was conducted, among others. Meanwhile, qualitative analysis will identify meanings and patterns through thematic analysis, such as mapping the strategies for the sustainability of IPE, through the categorization of data, depending on the findings of the research.

A qualitative and quantitative synthesis and a narrative of the data extracted from the selected studies will be developed to describe the characteristics of the discussions about sustainability and the actions or strategies (or both) of IPE, thus answering the research questions.

The information will be presented in tables, compiling the main results, conclusions, and implications.

### Consultation With Stakeholders

It is intended to introduce the preliminary results to 5 stakeholders in the field of IPE in such a way that they can analyze and identify any gaps that may be present. This step will help expand knowledge through new evidence not identified in the review, making it possible to develop strategies and directions for future studies [22].

To this end, the stakeholders will be sent an email with an informed consent, an electronic spreadsheet with the preliminary results, as well as a request for appraisal. Stakeholders will not be identified, and the authors will ask for an appraisal of the results and possible new fields or evidence, such as suggestions for additional articles or data that could be extracted from the studies.

### Ethical Considerations

This study will comply with the ethical principles of research with human beings [23]. It was therefore submitted to and approved by the Research Ethics Committee of the Onofre Lopes University Hospital of the Federal University of Rio Grande do Norte, under Protocol 6.483.476.

## Results

To this end, this study was registered with the Open Science Framework (OSF) on February 8, 2024. The database search step took place on March 22, 2024. In April and May 2024, the duplicate studies were excluded. In June 2024, the protocol was submitted to the journal. From July to November 2024, the selection of studies will be carried out. In December 2024, data extraction and tabulation will take place, as well as consultation with stakeholders. In January 2025, the review will be submitted to a scientific journal.

The results of this review will be shared through publications in renowned, peer-reviewed, and open access journals of great importance to public health and education, favoring and expanding the dissemination of knowledge to the scientific community. Any changes to this protocol will be reported appropriately in the record made in the OSF and in the final publication, including dates and justifications.

## Discussion

### Expected Findings

This protocol will guide this scoping review to identify discussions on the sustainability of IPE and map its actions or strategies (or both). This is similar to what was observed in a study conducted in 2021 [24], which presents enablers for the introduction of collaborative competence in the curriculum using an interprofessional framework. The study points out, based on previously developed experiences, that teamwork along with the definition of clear goals and strategic plans commonly increases the acceptance of team-based approaches among faculty and students. In this process, change management and communication skills are considered fundamental to this formula for success. Thus, awareness and a positive attitude toward the change process among faculty and students, through training on the attitudes, knowledge, and skills necessary to work efficiently with other professionals and provide safe, high-quality patient care, should be regarded as a key step during implementation.

Mapping this information will help decision makers, managers, teachers, facilitators, and students to implement, maintain, and develop IPE. It should be noted that vocational training is currently at a crucial moment for potential changes [7].

In addition, this review will shed light on existing knowledge gaps and the current state of research, which could support future research, programs, and policy responses to foster collaboration and interprofessional practice and, consequently, improve the quality of user care. In this way, the results of this review may support the development and implementation of more robust studies, such as systematic reviews, in addition to assisting in the development of primary studies, using other methodologies, thus expanding the results of studies already developed on IPE.

All the steps of the scoping review favor its transparency and allow it to be methodologically replicated according to the principles of open science, reducing the risk of bias and duplication of data.

It is noteworthy that the results will be disseminated to the academic community through scientific journals, scientific events, and other relevant public health communication channels.

### Limitations

This protocol guides the development of the scoping review. Nonetheless, it may not cover all the pertinent literature. Thus, studies indexed in different databases may not be traced in the searches. In addition, the limitation of language to English, Portuguese, and Spanish for the inclusion of studies may not locate some publications. Furthermore, the use of descriptors and search terms in English and Portuguese may also be a limitation for this study. However, the study will present the main results in the area, addressing the proposed objective.

### Conclusion

This study protocol introduces the main methodological steps that will be followed, so that the review will synthesize the main strategies and actions for IPE sustainability, the definitions and institutions that develop or foster IPE, as well as the main recommendations for the area under study. This knowledge will enable researchers, managers, teachers, students, and political decision makers to better understand what can be done in terms of implementing, maintaining, and developing IPE.

## Appendix

Multimedia Appendix 1 Search strategy for MEDLINE (PubMed). Research conducted in March 2024.

## Abbreviations

IPE: interprofessional education
JBI: Joanna Briggs Institute
MeSH: Medical Subject Headings
OSF: Open Science Framework
PRISMA-ScR: Preferred Reporting Items for Systematic Reviews and Meta-Analyses Extension for Scoping Reviews

## Data Availability

The spreadsheet with the consolidated data will be deposited in the Open Science Framework, so that the entire dataset on which the paper’s conclusions are based will be available to readers.
