# Supplementary material for: Sustainability of Interprofessional Education: Protocol for a Scoping Review
Source: JMIR Res Protoc. 2024 Dec 9;13:e60763. doi: 10.2196/60763 (PMC11667127; doi:10.2196/60763)
Supplement: Multimedia Appendix 1 [file resprot_v13i1e60763_app1.docx]

**Multimedia Appendix 1. Complete strategy for the search in Medline/Pubmed.**

| **Search** | **Query** | **Records retrieved**  (Mar 18, 2024) |
| --- | --- | --- |
| #1 | (((((((((((((((((((((("Interprofessional Education"[MeSH Terms]) OR ("Interprofessional Education"[Title/Abstract])) OR ("Education, Interdisciplinary"[Title/Abstract])) OR ("interdisciplinary education"[Title/Abstract])) OR ("Education, Interprofessional"[Title/Abstract])) OR ("cross-disciplinary education"[Title/Abstract])) OR ("cross-disciplinary studies"[Title/Abstract])) OR ("inter-disciplinary education"[Title/Abstract])) OR ("inter-disciplinary studies"[Title/Abstract])) OR ("interdisciplinary studies"[Title/Abstract])) OR ("multi-disciplinary education"[Title/Abstract])) OR ("multidisciplinary education"[Title/Abstract])) OR ("trans-disciplinary education"[Title/Abstract])) OR ("trans-disciplinary studies"[Title/Abstract])) OR ("transdisciplinary education"[Title/Abstract])) OR ("transdisciplinary studies"[Title/Abstract])) OR ("interdisciplinary education"[Title/Abstract])) OR ("Interdisciplinary Placements"[Title/Abstract])) OR ("Collaborative Learning"[Title/Abstract])) OR ("Collaborative Learnings"[Title/Abstract])) OR ("Interprofessional learning"[Title/Abstract])) OR (IPECP[Title/Abstract])) OR (IPE[Title/Abstract]) | 8,806 |
| #2 | ((Sustainability[Title/Abstract]) OR ("Sustainabilities, Program"[Title/Abstract])) OR ("Sustainability, Program"[Title/Abstract]) | 49,866 |
| #3 | #1 AND #2 | 144 |
| Language limit: English, Spanish, and Portuguese. | |  |
